# Segmental instrumentation in spinal infections: evaluating the role of metal implants in treatment outcomes

**DOI:** 10.25122/jml-2026-0015

**Published:** 2026-04

**Authors:** Bogdan Șendrea, Radu Josanu, Bogdan Gabriel Voicu, Sebastian Mihai Văleanu, Andrei Tudorache, Romică Cergan

**Affiliations:** 1 Foisor Clinical Hospital of Orthopedics, Traumatology and Osteoarticular TB, Bucharest, Romania; 2 Carol Davila University of Medicine and Pharmacy, Bucharest, Romania

**Keywords:** spinal infection, metal implant, antibiotic therapy, intervertebral instability, HIV, human immunodeficiency virus, IV, intravenous, MRI, magnetic resonance imaging, NTM, non-tuberculous mycobacteria, ENT, ear, nose, and throat, SSI, surgical site infections, CRP, C-reactive protein, ESR, erythrocyte sedimentation rate, WBC, white blood cell, CT, computed tomography, TB, tuberculosis

## Abstract

Spinal infections comprise a heterogeneous group of disorders involving the spinal column and adjacent neural elements, including spondylitis, discitis, spondylodiscitis, meningitis, myelitis, spinal epidural abscess, and infectious polyradiculopathy. The use of spinal instrumentation in infected fields remains a debated topic, although recent evidence suggests it may be safe when combined with adequate debridement and antibiotic therapy. Our objective was to evaluate the impact of segmental instrumentation on clinical, laboratory, and microbiological outcomes in patients undergoing surgery for spinal infections. We conducted a retrospective study of 98 adult patients who underwent surgical treatment for spinal infections between 2016 and 2024. Patients were divided into instrumented and non-instrumented groups based on intraoperative decision-making. Clinical outcomes, inflammatory markers, reoperation rates, and hospital stay were analyzed. The results showed that instrumentation was performed in 86 patients, while 12 patients underwent surgery without fixation. Instrumented patients showed comparable infection resolution and clinical recovery despite more severe preoperative presentations. Mean hospital stay was 18.79 days in the instrumented group and 17.91 days in the non-instrumented group. Inflammatory markers improved consistently in the instrumented group. Microbiological confirmation was achieved in approximately half of the cases. In conclusion, segmental instrumentation appears safe in the surgical management of spinal infections when combined with thorough debridement and targeted antibiotic therapy. Clinical outcomes are primarily influenced by infection control rather than the avoidance of implants.

## Introduction

Spinal infections represent a heterogeneous group of conditions involving the vertebrae, intervertebral discs, and adjacent soft tissues. These infections, although relatively uncommon, are associated with significant morbidity and potential neurological complications. The pathophysiological entity was common, dating back to the Iron Age [1], with some records showing that as early as 5000 years ago, people in India were aware of spinal tuberculosis, calling it “Yakshama” [2], although Percival Pott first formally described spinal tuberculosis in 1779 [3]. Due to the increase in antibiotic use, infections, including spinal infections, have experienced a resurgence, posing increasing challenges for their treatment.

Infections of the spine are a significant morbidity factor, even in modern days, with an incidence between 1:100,000 and 1:250,000 in developed countries, twice as high in men [3], and a mortality rate of 2–4% [4]. The lumbar spine is by far the most common site for spinal infection (58%) [5]. Factors that increase the incidence of spinal infections are age >50 years, long-standing chronic illnesses that affect the immune system, cancer, and previous spinal surgeries.

Regarding the clinical findings, the patient presents with general symptoms like fever, nausea, malaise, and specific symptoms like pain, paresthesia, paralysis, and urinary incontinence. When it comes to imaging, vertebral endplate destruction and/or the presence of abscesses adjacent to the spinal area are suggestive [6].

The treatment of spinal infections should be tailored to the clinical presentation, severity, risk factors, pathogenesis, and location. One of the more severe risk factors, especially in non-tuberculous mycobacterial (NTM) infections, is the patient’s immunocompromised state [7]. Patients with previous spinal surgeries, as well as patients with a history of travel to endemic regions, pose a significant risk [8]. The pathogenesis of spinal infections involves three major pathways: hematogenous spread from a distant site of infection, direct inoculation following surgery (spinal, ear, nose, and throat [ENT], or abdominal), and contiguous spread from adjacent tissues (e.g., the aorta, esophagus, or abdominal and pelvic structures) [6]. The most common route is hematogenous, particularly in the setting of bacterial endocarditis, which has been reported in up to 30% of spinal infections [9].

The second most common path is through surgical site infections (SSI), which is the reason in 16% of cases. The risk factors for SSI include interbody implants, length of stay, type of procedure, and patient age and medical history [10].

Direct dissemination from contiguous tissue is possible, but rare. Tissues that have demonstrated the potential for dissemination include the aorta, esophagus, pelvic structures, and adjacent vertebral bodies [6,11].

For suspected spinal infection, the initial assessment includes inflammatory markers (C-reactive protein [CRP], erythrocyte sedimentation rate [ESR], and white blood cell count [WBC]), spinal imaging (preferably magnetic resonance imaging [MRI]), and two sets of blood cultures, with serological and genetic identification methods available for additional diagnostic information.

CRP elevation has been shown to have a high sensitivity concurrent with inflammatory processes, making it ideal for diagnosing spinal infections in a patient with suggestive symptomatology and MRI findings. A low CRP level does not automatically rule out a spinal infection. Microbiological cultures are routinely used to identify pathogens.

MRI of the spine is the most sensitive technique for diagnosis [12]. In case of MRI contraindications, CT (computed tomography) scans, bone scintigraphy, radionuclide scanning, or just plain X-rays can provide clues to the potential underlying pathology.

When two blood cultures are positive, the diagnosis is considered established. In other cases, a biopsy is required to confirm the diagnosis, with a recommended 14-day antibiotic washout period for patients who have previously received antimicrobial therapy [13]. In patients presenting with severe sepsis or neurological deficits, antibiotic therapy should be initiated promptly.

The most common differential diagnosis is represented by neoplastic processes. Clinically, both conditions typically present with an insidious onset of back pain, often worsening at night. MRI is usually the preferred modality for differentiation. Disc involvement is uncommon in tumors but is a characteristic finding in infections. Additionally, edema associated with infectious processes tends to obscure fat planes, whereas neoplastic lesions generally preserve them [14].

Non-operative treatment comprises empirical or targeted antibiotic therapy, bed rest for 1-2 weeks, physical therapy, and spinal immobilization with adapted orthotic devices [15]. Empirical antibiotic therapy typically includes a third-generation cephalosporin or fluoroquinolone combined with clindamycin or vancomycin. If the patient’s condition worsens after 6 weeks of conservative treatment, surgical treatment is recommended.

Surgical intervention is mandatory in cases of spinal cord compression associated with neurological impairment, as deficits caused by spinal infections may lead to permanent neurological damage within as little as 24 hours after onset [16]. The main principles of surgery include thorough debridement, preservation of adequate blood flow, and ensuring spinal stability, with restoration when necessary [17].

The surgical techniques used in the management of spinal infections include laminectomy, multi-segmental decompression, corpectomy, vertebral reconstruction, and posterior transpedicular segmental instrumentation. While a laminectomy alone is used in liquid space-occupying lesions, the other procedures are used for solid space-occupying lesions. If needed, a staged operation can be performed, with the initial surgery focused on debriding the infection site, while subsequent surgical interventions may focus on stabilizing the affected segment. In one-stage procedures, autologous bony grafts are sometimes preferred, but not exclusively used [18].

## Material and methods

This retrospective study included 98 adult patients (>18 years) who underwent surgical treatment for spinal infections at the “Foișor” Clinical Hospital in Bucharest between 2016 and 2024. During the initial consult, the following variables were recorded: neurological status (Frankel grading scale), age and sex, comorbidities and immunosuppressive conditions, laboratory values (CRP, ESR, WBC), and imaging findings. The treatment protocol consisted of empirical intravenous antibiotics initiated upon suspicion of infection, followed by biopsy, with antibiotic therapy adjusted accordingly based on culture results. The mean duration of antibiotic therapy was 6–8 weeks (IV followed by oral therapy). Based on the amount of spinal instability, the surgical team decided whether to perform a segmental instrumentation. Patients were discharged after satisfactory post-operative clinical and laboratory evolution. The study was conducted in accordance with the World Medical Association (WMA) Declaration of Helsinki.

## Results

**Figure 1:**
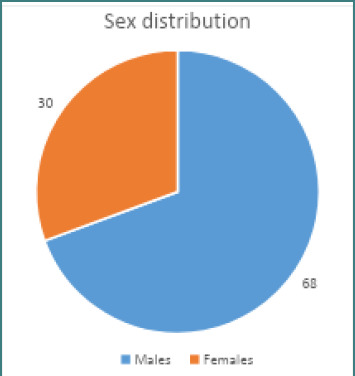
Sex distribution

**Figure 2:**
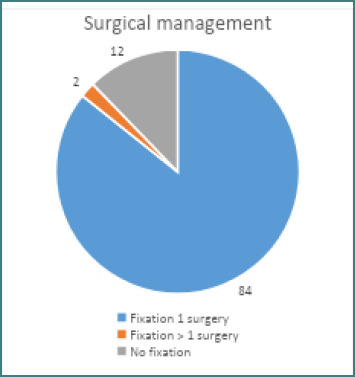
Surgical management

A total of 98 patients met the inclusion criteria. Of these, 68 were men, and 30 were women, with a mean age of 56 years in the male group and 57.5 years in the female group (Figure 1). Regarding surgical management, 84 patients required a single surgical intervention to achieve full clinical recovery, while two patients underwent more than two surgeries. 12 patients did not require fixation, as they presented without neurological deficits at admission (Figure 2).

Regarding comorbidities, 43 patients had immunosuppressive conditions, while isolated cases presented with multiple concurrent conditions; however, the distribution of intermediate categories (one or two conditions) was not consistently documented.

At admission, 47 patients showed no suspicion of infection, while 51 were suspected of having tuberculosis (TB). Additionally, 12 patients had a previously confirmed TB infection, and two patients had other confirmed infections. These categories were not mutually exclusive and may overlap.

**Figure 3:**
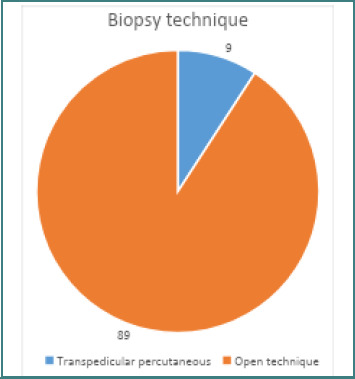
Biopsy technique

**Figure 4:**
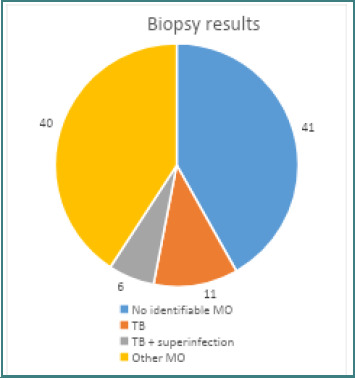
Biopsy results

All 98 patients underwent biopsy, of which nine were performed using a percutaneous transpedicular approach and 89 via an open technique (Figure 3). Microbiological analysis revealed no identifiable organism in 41 cases. Tuberculosis was confirmed in 11 cases, while six cases demonstrated TB with a concomitant superinfection. In 40 cases, the identified pathogen differed from the initial clinical suspicion (Figure 4). There was a significant discrepancy between suspected and confirmed diagnoses (*P* < 0.01), highlighting the importance of tissue diagnosis.

Prior to the availability of biopsy results, 27 patients were initiated on the hospital’s tuberculosis (TB) treatment protocol based on preoperative and intraoperative assessments. Perioperatively, 61 patients received a standard antibiotic regimen consisting of vancomycin and cefuroxime, while a subset received individualized antibiotic therapy guided by clinical parameters; however, complete data were not available for all patients.

Among patients undergoing spinal fusion, improvements in inflammatory markers (ESR, CRP, WBC) were observed in 27 cases. In contrast, only two patients in the non-fusion group demonstrated similar improvements.

Patients treated with spinal fusion had a mean hospitalization duration of 18.79 days (range 10–89 days), compared to 17.91 days (range 10–25 days) in non-operatively managed patients. The difference was not statistically significant (*P* = 0.67).

Neurological recovery was generally favorable. One patient improved from Frankel grade B to D, while the majority of the remaining patients achieved full recovery. One postoperative death was recorded due to cardiorespiratory failure. Among confirmed TB cases, nearly all patients required spinal fusion, with only one case managed without surgical stabilization, indicating a strong association between TB diagnosis and surgical intervention (*P* < 0.01).

## Discussion

In this single-center cohort of 98 adults treated surgically for spinal infections between 2016 and 2024, segmental instrumentation was not associated with clinical or laboratory evidence of persistent infection, despite being used in more unstable and neurologically severe cases. Instrumented patients had only a minimally longer hospital stay than non-instrumented patients (18.79 vs. 17.91 days) and showed consistent improvement in ESR, CRP, and WBC, indicating that metallic implants did not promote ongoing sepsis when combined with adequate debridement and targeted antibiotic therapy. Robinson *et al*. reported excellent results for functional healing and laboratory dynamic values whilst treating spinal infections with intervertebral cages, with a mean follow-up of 36 months [19].

The demographic profile of our cohort, with a predominance of older male patients and frequent comorbidities, mirrors contemporary epidemiological data on spondylodiscitis and reflects the known risk factors of age, chronic systemic disease, and immunosuppression. The high proportion of suspected or confirmed TB cases corresponds to the endemic context and underscores TB as a major cause of spinal infection in regions with socio-economic disparities.

Nearly half of the biopsies did not yield a causative organism despite a large number of open procedures, highlighting the persistent difficulty of microbiological confirmation in routine practice. Prior antibiotic exposure and limitations of culture techniques likely contributed to these results, while the frequent discordance between admission suspicion and biopsy findings underlines the importance of obtaining tissue samples to guide therapy. The identification of TB with concomitant superinfection in several cases further supports comprehensive microbiological work-up whenever feasible. KY Lee highlights relevant factors concurrent with the current study’s findings regarding the limitations of microbial identification, as well as clinical and laboratory studies [20].

Patients treated with fusion experienced favorable neurological outcomes, including improvement from Frankel B to Frankel D in one severe case and full or near-full recovery in the remainder. Given that instrumented cases typically represented more extensive disease and greater instability, the absence of excess complications or markedly prolonged hospitalization argues against the assumption that implants inherently maintain or exacerbate infection. Our findings are consistent with contemporary evidence that, when meticulous debridement and prolonged, culture-directed antibiotics are used, instrumentation in acute spinal infection is safe and may facilitate earlier mobilization and deformity prevention, as Yagdiran *et al*. found in their study [21].

This study is limited by its retrospective, single-center design, relatively small sample size, and incomplete long-term imaging follow-up, which restricts generalizability and detailed pathogen-specific analysis. Future prospective multicenter studies integrating advanced molecular diagnostics and standardized follow-up are needed to further clarify recurrence risk, hardware-related complications, and functional outcomes in instrumented versus non-instrumented spinal infections.

## Conclusion

Considering the severity of the cases requiring fusion and the fact that implants have historically been considered debatable in the context of infection treatment, it is noteworthy that patients undergoing spinal fusion had, on average, only a 0.88-day longer hospital stay. While the maximum length of stay varied (14 patients had stays longer than 25 days, the maximum for non-surgical cases), these findings suggest that, at the very least, spinal fusion stabilizes the spine without serving as a favorable site for infection. With follow-up evaluations showing near-unanimous complete neurological recovery, it can be concluded that successful infection resolution depends primarily on adequate debridement, accurate microbiological identification, and appropriate postoperative treatment, rather than on avoiding the use of metallic implants, which may otherwise hinder patient recovery.
